# Atypical Autoimmune Encephalitis: Diagnostic Challenges and Therapeutic Insights From a Case Series

**DOI:** 10.7759/cureus.82384

**Published:** 2025-04-16

**Authors:** Thummalagunta Prathyusha, Gowtham Ambati, Abhijathya Chinta, Pavan Gowda, Chandraditya Bole

**Affiliations:** 1 General Medicine, All India Institute of Medical Sciences, Mangalagiri, Mangalagiri, IND; 2 Internal Medicine, All India Institute of Medical Sciences, Mangalagiri, Mangalagiri, IND

**Keywords:** anti-nmda receptor encephalitis, autoantibodies, autoimmune encephalitis, intravenous immunoglobulins (ivig), paraneoplastic encephalitis syndromes, rasmussen's encephalitis, status epilepticus (se)

## Abstract

Encephalitis syndromes are acute neurological emergencies characterized by altered sensorium, seizures, movement disorders, and neuropsychiatric manifestations. Autoimmune encephalitis (AE), although rare, necessitates prompt recognition and intervention to reduce morbidity and mortality. This study aimed to examine the clinical features, laboratory findings, radiological characteristics, and treatment outcomes of patients diagnosed with autoimmune encephalitis. This case series presents six patients diagnosed with autoimmune encephalitis and admitted to the Department of General Medicine at the All India Institute of Medical Sciences (AIIMS), Mangalagiri, between January 2024 and January 2025. The mean age of onset was 30.4 years. The cohort included two cases of anti-N-methyl-D-aspartate (anti-NMDA) receptor encephalitis, one case with both myelin oligodendrocyte glycoprotein (MOG) and anti-NMDA receptor antibodies, one case of seronegative autoimmune encephalitis (SNAE) diagnosed based on magnetic resonance imaging (MRI) findings, one case of Rasmussen's encephalitis (RE), and one case of paraneoplastic autoimmune encephalitis (PAE) associated with lung malignancy. This study provides a comprehensive overview of clinical presentations, treatment approaches, and patient outcomes. Early diagnosis and timely initiation of immunosuppressive therapy are crucial for improving patient outcomes. Diagnosis relies on clinical assessment, neuroimaging, and antibody testing, with seronegative cases requiring a high degree of clinical suspicion for appropriate management.

## Introduction

Autoimmune encephalitis (AE) is a group of brain disorders caused by the immune system attacking nerve cells, leading to various mental and neurological symptoms. It involves autoantibodies that disrupt brain function and cause inflammation. AE is classified based on the type of antibody against cell surface (AE) or intracellular targets (paraneoplastic encephalitis). Other similar conditions include Rasmussen's encephalitis (RE) and seronegative AE. AE is now recognized as a major cause of non-infectious encephalitis, making early diagnosis and treatment essential for better outcomes.

The global prevalence of AE is estimated to be approximately 13.7 cases per 100,000 persons per year, with anti-N-methyl-D-aspartate (anti-NMDA) receptor encephalitis being the most common subtype. In India, epidemiological data on AE are limited, but available studies suggest an increasing number of reported cases due to heightened awareness and improved diagnostic capabilities [1-3]. As per studies, anti-NMDA receptor encephalitis is the most well-characterized and frequently encountered subtype, with an estimated number of cases of 1.5 per million people per year, with a median age of 21 years (range: 8 months to 85 years) [4,5]. The prevalence of specific neural autoantibody biomarkers among the definite autoimmune encephalitis category was as follows: glutamic acid decarboxylase-65 (GAD65), 1.9 per 100,000; unclassified neural autoantibody, 1.4 per 100,000; myelin oligodendrocyte glycoprotein (MOG), 1.3 per 100,000; leucine-rich glioma-inactivated protein 1 (LGI1), 0.7 per 100,000; collapsin response-mediator protein-5 (CRMP5), 0.7 per 100,000; N-methyl-D-aspartate (NMDA) receptor, 0.6 per 100,000; anti-neuronal nuclear antibody-2 (ANNA-2/anti-Ri), 0.6 per 100,000; and glial fibrillary acidic protein-α (GFAPα), 0.6 per 100,000 [6].

The diagnosis of autoimmune encephalitis relies on a combination of clinical assessment, neuroimaging, cerebrospinal fluid (CSF) analysis, and autoantibody testing. Magnetic resonance imaging (MRI) often reveals hyperintensities in the limbic system, particularly in cases of limbic encephalitis (LE). Analysis of CSF may show pleocytosis, elevated protein levels, and the presence of specific autoantibodies.

Seronegative autoimmune encephalitis (SNAE) is diagnosed in patients meeting the clinical and radiological criteria for AE but lacking identifiable autoantibodies. The 2016 diagnostic criteria aid in classifying definite and probable AE, particularly in seronegative cases, as described in a study proposed by Graus et al. [7]. Despite the absence of known antibodies, patients often respond favorably to corticosteroids, intravenous immunoglobulin (IVIG), or plasma exchange (PLEX), reinforcing the autoimmune basis of the disease. Paraneoplastic autoimmune encephalitis (PAE) is associated with underlying malignancies, most commonly small cell lung cancer, ovarian teratomas, and thymomas. According to studies, the number of clinically relevant antibody reactivities as markers of paraneoplastic disorders has grown at the rate of about one per year [8]. It was subsequently learned that the clinical syndrome is correlated with the associated autoantibodies. Several classes of antibodies have been described in association with LE [9]: the classical onconeuronal antibodies that are directed against intracellular antigens and those directed against surface proteins (voltage-gated potassium channel (VGKC) complex, NMDA receptor (NMDAR), AMPA receptor (AMPAR), and γ-aminobutyric acid (b) (GABA (b))). It is mediated by onconeural antibodies such as Hu, Yo, and Ma2, which cross-react with neuronal antigens. Diagnosing AE early is key to helping patients recover better. Delaying treatment can lead to worse brain function, long-term disability, and a higher risk of death. Starting immunotherapy on time can ease symptoms, prevent problems, and support recovery.

This case series examines six patients diagnosed with autoimmune encephalitis, elucidating their clinical presentations, diagnostic evaluations, treatment regimens, and outcomes over a span of one year. The results underscore the significance of prompt recognition, personalized therapy, and sustained monitoring in effectively managing AE.

## Materials and methods

This case series includes six patients diagnosed with autoimmune encephalitis and admitted to the All India Institute of Medical Sciences (AIIMS), Mangalagiri, between January 2024 and January 2025. Informed consent was obtained from all patients or their legal guardians before inclusion in the study.

Inclusion and exclusion criteria

Patients fulfilling the 2016 criteria proposed by Graus et al. [7] for definite or probable autoimmune encephalitis (Table 1), based on clinical-radiological and/or serological findings with follow-up of at least three months post-discharge, after excluding other causes of encephalitis (i.e., infectious and metabolic (via CSF routine tests, viral polymerase chain reaction (PCR), culture and sensitivity, adenosine deaminase levels, and serum electrolytes)), were included in the study. Additionally, patients who received treatment at external facilities or had incomplete medical records that limited comprehensive evaluation were excluded from the final dataset.

**Table 1:** 2016 diagnostic criteria for autoimmune encephalitis. CNS: central nervous system, CSF: cerebrospinal fluid, WBC: white blood cell count, MRI: magnetic resonance imaging, FLAIR: fluid-attenuated inversion recovery, EEG: electroencephalogram, AE: autoimmune encephalitis, NMDAR: N-methyl-D-aspartate receptor, LGI1: leucine-rich glioma-inactivated protein 1, CASPR2: contactin-associated protein-2, AMPAR: alpha-amino-3-hydroxy-5-methyl-4-isoxazolepropionic acid receptor, GABA: γ-aminobutyric acid Source: Graus et al. [7]

Diagnostic level	Criteria
Possible autoimmune encephalitis	Subacute onset (<3 months) of working memory deficits, altered mental status, or psychiatric symptoms; at least one of the following: new focal CNS findings, new-onset seizures, CSF pleocytosis (WBC > 5/mm³), and MRI suggestive of encephalitis; and reasonable exclusion of alternative causes
Probable autoimmune encephalitis (e.g., limbic encephalitis)	Subacute onset of memory issues, confusion, psychiatric symptoms, or seizures; bilateral abnormalities on T2/FLAIR MRI in medial temporal lobes; at least one of the following: CSF pleocytosis and EEG showing temporal lobe abnormalities; and exclusion of other causes (infectious, metabolic, etc.)
Definite autoimmune encephalitis	Meets criteria for possible or probable AE; detection of specific AE-related autoantibodies (e.g., anti-NMDAR, LGI1, CASPR2, AMPAR, and GABA A/GABA B receptors)

Data collection

For every patient, a thorough clinical assessment was conducted, encompassing history-taking and neurological examination. Demographic particulars such as age, gender, and initial symptoms were documented. Laboratory analyses comprised standard blood tests, assessment of inflammatory markers, cerebrospinal fluid (CSF) examination, and autoantibody testing, where applicable.

Neuroimaging and diagnosis

All patients underwent an MRI of the brain with contrast, with T2/fluid-attenuated inversion recovery (FLAIR) sequences evaluated for hyperintensities suggestive of encephalitis. An electroencephalogram (EEG) was performed in cases presenting with seizures. The diagnosis of autoimmune encephalitis was made based on a combination of clinical, imaging, and serological findings. For seronegative cases, MRI and clinical correlation were key factors in diagnosis.

Treatment protocol

All patients received high-dose intravenous methylprednisolone (IVMP) as first-line therapy. In severe or refractory cases, additional immunotherapies, including IV immunoglobulins (IVIG), plasma exchange (PLEX), and rituximab, were administered. Symptomatic management, including antiepileptic drugs and supportive care, was provided as required.

Follow-up and outcome assessment

Patients were followed up for at least three months post-discharge to assess neurological recovery, seizure control, and functional improvement. Clinical outcomes were categorized based on symptom resolution, persistence of neurological deficits, and mortality in the cases measured using the modified Rankin Scale (mRS) and mortality rates.

## Results

Clinical presentation

Cases 1-3: Anti-NMDA Receptor Encephalitis

Anti-NMDA receptor encephalitis manifests with a diverse range of neuropsychiatric symptoms, as demonstrated by three distinct cases.

Case 1: A 63-year-old female patient initially displayed alterations in behavior, restlessness, and disorientation, which swiftly progressed to seizures, autonomic dysfunction, and altered consciousness, necessitating ventilatory and critical care. Remarkably, there was evidence of bowel and bladder incontinence, underscoring significant autonomic involvement. MRI results revealed T2/FLAIR hyperintensities in the right greater than the left temporal lobe and hippocampus, while cerebrospinal fluid (CSF) analysis confirmed the presence of anti-NMDA receptor antibodies. Due to unresponsive seizures, the patient necessitated mechanical ventilation. Prompt initiation of immunotherapy, comprising IV methylprednisolone, rituximab, and plasma exchange, resulted in gradual amelioration. A tracheostomy was conducted in view of prolonged ventilatory support and not being able to maintain ventilation without assistance to prevent infections, and the patient was discharged on antiepileptic medications, such as levetiracetam, valproate, and phenytoin (Table 2). Challenges in managing the patient included advanced age, obesity, and obstructive sleep apnea (OSA). During follow-up, successful closure of the tracheostomy was achieved, and the patient regained the ability to sit and walk with assistance. She continued on antiepileptic drugs and supportive therapy, with her most recent follow-up appointment being five months prior.

**Table 2:** Demographic details, clinical presentation, imaging, investigations, treatment modalities, and outcomes of the six patients diagnosed with autoimmune encephalitis. MRI: magnetic resonance imaging, IVIG: intravenous immunoglobulin, PLEX: plasma exchange, AE: autoimmune encephalitis, NMDA: N-methyl-D-aspartate

Demographics	Case 1	Case 2	Case 3	Case 4	Case 5	Case 6
Age (years)	63	26	17	20	43	28
Sex	Female	Female	Female	Female	Female	Male
Initial symptoms	Bowel and bladder incontinence	Delusion of grandeur, extrapyramidal symptoms	Fever, altered sensorium	Involuntary movements of the left upper and lower limbs	Cognitive dysfunction, refractory seizures	Fever, altered sensorium, recurrent seizures, respiratory distress since 2 days
Psychological and behavioral abnormalities	Altered sensorium	Delusion of grandeur	Involuntary laugh/cry	-	-	+
Epileptic seizure	+	+	+	+	+	+
Cognition impairment	+	+	+	-	+	-
Mechanical ventilation	+	+	-	-	-	+
Presence of MRI findings	+	+	+	+	+	+
Cancer screening	-	-	-	-	-	Lung adenocarcinoma
Treatment	Glucocorticoid, rituximab	Glucocorticoid, rituximab/IVIG, PLEX	Glucocorticoid, rituximab/IVIG, PLEX	Glucocorticoid, rituximab	Glucocorticoid, rituximab	Glucocorticoid, rituximab, malignancy management
Outcome	Improved with a modified Rankin Scale score of 3	Neurological deficit present with a modified Rankin Scale score of 4	Completely recovered with a modified Rankin Scale score of 1	Intensity of movement decreased with a modified Rankin Scale score of 3	Completely recovered with a modified Rankin Scale score of 2	Succumbed to malignancy after 4 months with a modified Rankin Scale score of 6
AE antibody testing	Anti-NMDA receptor antibody +	Anti-NMDA receptor antibody +	Anti-NMDA receptor antibody +	-	-	-

**Figure 1:**
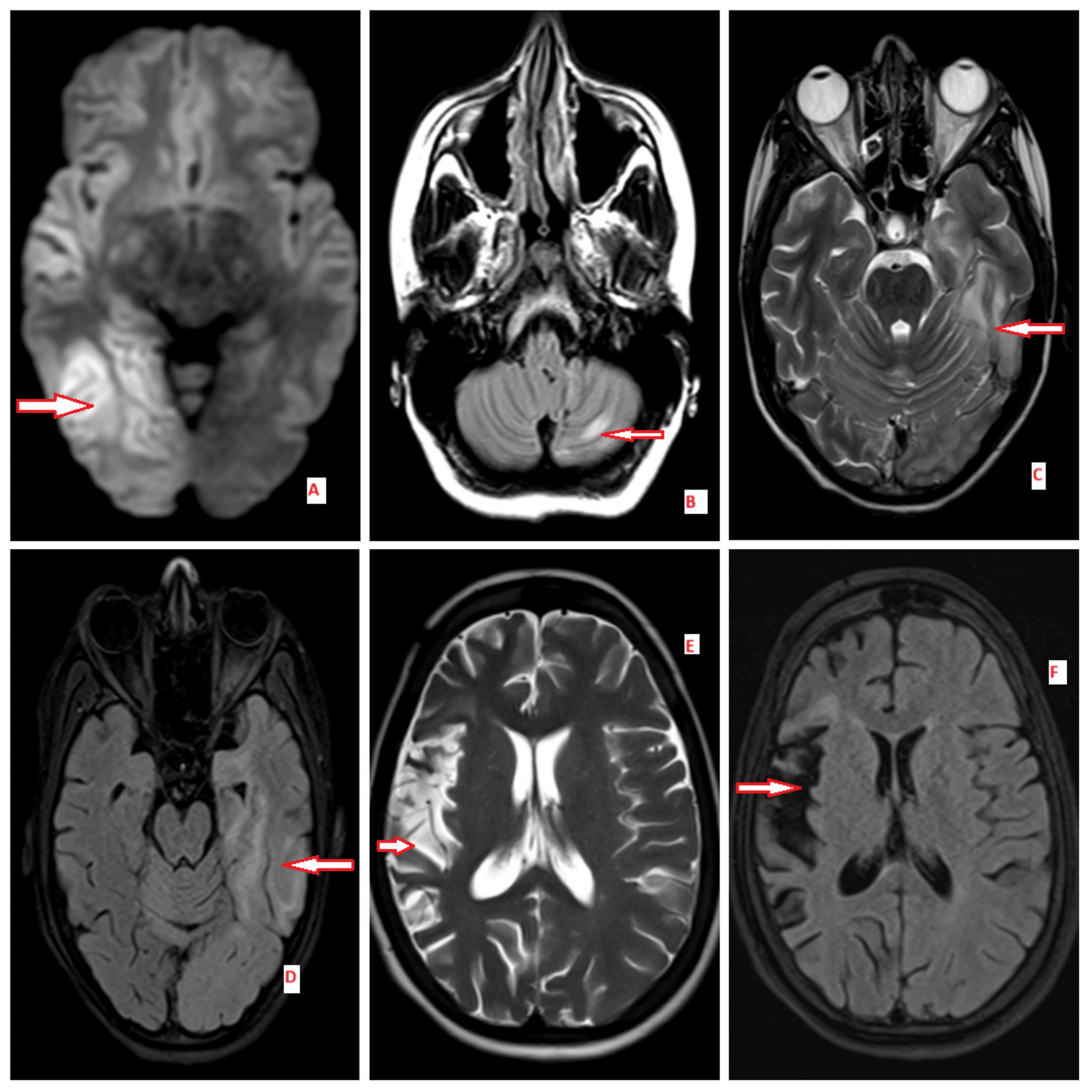
A and B MRI images (Case 2): T2/FLAIR hyperintensities in the right > left temporal and hippocampus. C and D MRI images (Case 3): T2/FLAIR hyperintensities with diffusion restriction in the left temporal and occipital area. E and F MRI images (Case 4): T2/FLAIR right hippocampus and cerebral hemisphere atrophy suggestive of Rasmussen's encephalitis. Red arrow: lesion MRI: magnetic resonance imaging, FLAIR: fluid-attenuated inversion recovery

Case 2: A 26-year-old female patient presented with a sudden onset of altered behavior, psychiatric symptoms such as delusions of grandeur, and severe agitation, which then progressed to refractory status epilepticus. Imaging studies revealed T2/FLAIR hyperintensities with diffusion restriction in the right temporal and hippocampal regions (Figure 1A, 1B). The cerebrospinal fluid analysis confirmed the presence of anti-NMDA receptor antibodies, while other findings were inconclusive, and infectious and autoimmune workups were negative. She was initially intubated and put on mechanical ventilation due to respiratory failure in the form of increased respiratory rate, decreasing SPO2, and increased risk of aspiration in view of status epilepticus. Subsequently, she was commenced on immunotherapy, which included five doses of IV methylprednisolone, five doses of IVIG, plasma exchange, and two doses of rituximab (Table 2). The patient's clinical course was complicated due to recurrent infections and Stevens-Johnson rash. Furthermore, her clinical course was marred by a cardiac arrest due to sepsis with pneumonia, necessitating resuscitation with cardiopulmonary resuscitation (CPR). Following stabilization, a tracheostomy was performed to facilitate prolonged ventilatory support. Post-stabilization, she was prescribed three antiepileptic medications and underwent extensive supportive physiotherapy. Upon discharge, she continued to rely on a tracheostomy tube for room air intake and was confined to a wheelchair. However, there were indications of neurological recovery as she exhibited the ability to stand with assistance, consume semisolid and liquid nourishment, and articulate a few words, signaling a gradual albeit limited functional improvement.

Case 3: A 17-year-old patient presented with an acute febrile illness, leading to altered sensorium and recurrent seizures over a span of 15 days. Despite a history of a positive urine pregnancy test, the patient experienced an incomplete abortion. Notably, she displayed atypical symptoms, such as episodes of incongruous laughter and crying, prompting suspicion of autoimmune encephalitis. Imaging revealed T2/FLAIR hyperintensities with diffusion restriction in the left temporal and occipital regions (Figure 1C, 1D). Although her symptoms initially resembled those of viral encephalitis, a viral PCR test yielded negative results. Instead, the presence of anti-NMDA and anti-MOG antibodies confirmed an overlap syndrome. The patient exhibited a positive response to a treatment regimen involving steroids, IVIG, plasma exchange, and rituximab (Table 2). Subsequently, she underwent a medical termination of pregnancy for incomplete abortion. Following treatment, her condition improved significantly, leading to her discharge with a prescription for two antiepileptic medications. At the time of follow-up, the patient remained seizure-free, with no lingering complications.

Case 4: Rasmussen's Encephalitis

A 20-year-old woman presented with progressive focal seizures and choreiform movements, characterized by involuntary movements of the left upper and lower limbs. The neurological symptoms gradually worsened over time, significantly impacting her daily activities. A brain MRI revealed T2/FLAIR hyperintensities involving the right hippocampus and cerebral hemisphere in frontoparietal areas (Figure 1E, 1F), findings consistent with Rasmussen's encephalitis. Unlike anti-NMDA receptor encephalitis, the CSF analysis was negative for autoantibodies, posing a diagnostic challenge and necessitating a clinical and radiological approach for confirmation.

The patient was initiated on high-dose intravenous methylprednisolone (IVMP) for five days, followed by two doses of rituximab. While she demonstrated partial improvement in movement symptoms, seizure control remained a concern, necessitating continued medical management and close monitoring. Additionally, an incidental finding of a Breast Imaging Reporting and Data System (BI-RADS) IV left breast outer quadrant lesion required ongoing oncological evaluation, further complicating long-term management (Table 2).

On follow-up, there was a reduction in the intensity of involuntary movements, although residual symptoms persisted. Her last in-person visit was four months ago, and subsequent telephonic consultations were conducted to assess her condition. She was counseled regarding the need for further treatment and reassessment, emphasizing the importance of regular follow-ups to monitor both her neurological status and breast lesion evaluation.

Case 5: Seronegative Autoimmune Encephalitis

A 43-year-old woman presented with intractable seizures persisting for one week, accompanied by cognitive impairment. Upon neurological assessment and brain MRI, T2/FLAIR hyperintensities were observed in the bilateral hippocampus and bilateral frontal lobes, indicative of limbic encephalitis. Despite the absence of known autoantibodies in the cerebrospinal fluid (CSF) analysis, posing a diagnostic conundrum in establishing an autoimmune origin, the clinical and radiological evidence strongly suggested an autoimmune inflammatory process.

The patient commenced treatment with high-dose intravenous methylprednisolone (IVMP) for five days, followed by two doses of rituximab (Table 2). Subsequently, she exhibited a favorable response to immunotherapy, manifesting significant enhancements in seizure management and cognitive functionality. Upon discharge, she was prescribed a dual antiepileptic regimen, achieving sustained seizure remission.

During subsequent evaluations, the patient maintained a seizure-free status with no residual neurological impairments or complications. The primary obstacle in her care lay in the absence of autoantibody indicators, necessitating a clinical diagnosis and empirical immunotherapy. Her case underscores the significance of identifying seronegative autoimmune encephalitis and promptly initiating treatment despite the lack of definitive serological affirmation.

Case 6: Paraneoplastic Autoimmune Encephalitis

A 28-year-old man presented with fever, altered consciousness, recurrent seizures, and respiratory distress over a span of two days. Upon neurological assessment and MRI examination, T2/FLAIR hyperintensities with diffusion restriction were noted in the left temporal and occipital regions, prompting suspicion of autoimmune encephalitis. Nevertheless, cerebrospinal fluid (CSF) analysis yielded negative results for established autoantibodies, necessitating further scrutiny.

Subsequent imaging studies unveiled an underlying lung adenocarcinoma, culminating in the diagnosis of paraneoplastic autoimmune encephalitis. As the patient's condition deteriorated, necessitating respiratory support, he was intubated due to respiratory failure (Table 2). Owing to the paraneoplastic origin of the condition, the patient was transferred to a specialized facility for the management of the carcinoma. Despite the application of intensive supportive measures and immunomodulatory therapy, the patient's health continued to decline, ultimately succumbing to oncological complications within a four-month period.

Table 3 provides a description of the modified Rankin Scale score.

**Table 3:** Modified Rankin Scale.

Score	Description
0	No symptoms
1	No significant disability, able to carry out all usual activities, despite some symptoms
2	Slight disability, able to look after own affairs without assistance but unable to carry out all previous activities
3	Moderate disability, requires some help but able to walk unassisted
4	Moderately severe disability, unable to attend to own bodily needs without assistance and unable to walk unassisted
5	Severe disability, requires constant nursing care and attention, bedridden, incontinent
6	Dead

## Discussion

Anti-NMDA receptor encephalitis

Anti-NMDA receptor encephalitis is more common in women, as reported by Samanta and Lui [10], a finding corroborated by our case series, where all affected patients were female. The initial presentation in the three cases varied widely, ranging from behavioral disturbances and psychiatric symptoms to refractory seizures and autonomic instability, consistent with observations by Samanta and Lui [10].

Case 1, an elderly patient, exhibited severe autonomic dysfunction, including bowel and bladder incontinence, illustrating the significant impact of the disease on the autonomic nervous system. In contrast, Case 2 was marked by acute psychiatric symptoms with delusions of grandeur and refractory status epilepticus, highlighting the profound neuropsychiatric involvement in younger adults. Case 3 mimicked viral encephalitis, with fever, altered sensorium, and inappropriate laughter and crying episodes, making early differentiation from infectious causes challenging. Notably, this patient also demonstrated positivity for anti-NMDA and anti-MOG antibodies, suggesting an overlap syndrome that further complicates disease management and prognosis.

The initial characterization of anti-NMDA receptor encephalitis was published by Dalmau et al. in 2007, who identified a previously unrecognized autoimmune syndrome linked to NMDA receptor antibodies and ovarian teratomas [11]. It is an autoimmune disorder associated with antibodies against the N-methyl-D-aspartate receptor subunit 1 (NR1) of the NMDA receptor. The disorder predominantly affects young adults and children, with a strong female preponderance, particularly in cases associated with ovarian teratomas. Neuroimaging findings across the cases demonstrated T2/FLAIR hyperintensities predominantly affecting the temporal and hippocampal regions, aligning with typical patterns observed in autoimmune encephalitis, as described in a cohort study by Khatib et al., which found a higher prevalence of temporal hyperintensities [12]. CSF analysis was pivotal in confirming the diagnosis. In Case 3, viral encephalitis was ruled out, reinforcing the necessity of antibody testing in suspected autoimmune encephalitis and demonstrating positivity to anti-NMDA receptor antibodies, aligning with findings that the sensitivity of NMDA receptor antibody testing is higher in CSF compared to serum, as reported by Wang et al. [13]. Early and aggressive immunotherapy, including corticosteroids, intravenous immunoglobulin (IVIG), plasma exchange, and rituximab, was employed in all cases, leading to varying clinical improvement. However, treatment was complicated in Case 2, which developed drug-induced Stevens-Johnson syndrome and hepatitis, underscoring the risks of immunomodulatory therapy, with recurrent infections and a cardiac arrest further complicating management.

Studies indicate that first-line treatments, such as corticosteroids, IVIG, and plasma exchange, result in clinical improvement in approximately 50% of cases, with second-line agents such as rituximab and cyclophosphamide being required in refractory cases. The prognosis is highly dependent on early diagnosis and intervention, with up to 80% of patients achieving significant recovery if treated early, whereas delayed treatment is associated with worse neurological outcomes and prolonged hospital stays.

Challenges in management included pre-existing conditions such as obesity and obstructive sleep apnea in Case 1. Prolonged mechanical ventilation and tracheostomy in Cases 1 and 2 highlighted the severity of the disease, and the prolonged course of recovery with residual motor and speech impairments persisted, necessitating long-term rehabilitation. Case 3 demonstrated the best outcome, with seizure-free status and no significant residual deficits, as described by Nosadini et al., who reported symptom-free disease in follow-up [14].

Given the spectrum of complications, a multidisciplinary approach involving neurology, psychiatry, critical care, and rehabilitation specialists is crucial to optimize patient outcomes. Furthermore, the co-occurrence of anti-NMDA and anti-MOG antibody positivity in Case 3 raises important questions about underlying immune mechanisms. The role of long-term rehabilitation, including physical and cognitive therapy, is increasingly recognized as vital for functional recovery, and long-term monitoring is required owing to a relapse rate of 10%-30%, as mentioned in the literature by Berek et al. in 2022 with long-term follow-up [15].

Despite advances in understanding this disease, challenges remain, particularly in resource-limited settings where antibody testing may not be readily available. Additionally, the lack of standardized diagnostic criteria and treatment guidelines underscores the need for further research to optimize management strategies.

Rasmussen's encephalitis

In contrast to other autoimmune encephalitides, Rasmussen's encephalitis (RE) typically lacks serological markers such as anti-NMDA or anti-LGI1, making the diagnosis reliant on clinical and radiological criteria. In our case, the patient presented with hemichorea and unilateral seizure, findings consistent with the diagnostic criteria for RE, as originally described by Rasmussen et al. [16]. Treatment strategies for RE include high-dose corticosteroids, IVIG, plasma exchange, and immunosuppressants such as rituximab or cyclophosphamide. In severe cases, surgical interventions such as functional hemispherectomy may be required, as discussed in the study by Varadkar et al. [17]. Our patient received IVMP and rituximab, with partial symptomatic improvement, although seizure control remained a challenge. Long-term follow-up is essential, particularly considering the incidental BI-RADS IV breast lesion requiring oncological evaluation. The outcome in the present case is comparable to the study by Bien et al. [18].

Seronegative autoimmune encephalitis

Seronegative autoimmune encephalitis (SNAE) refers to cases presenting with clinical and radiological features of autoimmune encephalitis but lacking detectable neuronal autoantibodies in CSF or serum. The 2016 diagnostic criteria proposed by Graus et al. for SNAE focus on disorders localized to the limbic system and require the presence of bilateral T2-weighted fluid-attenuated inversion recovery (FLAIR) hyperintensities restricted to the mesiotemporal lobes on brain MRI [7]. Notably, a positive antibody status is not mandatory for a diagnosis of definite autoimmune LE, as bilateral mesiotemporal hyperintensities are considered highly specific for an immune-mediated disorder with subacute onset of neurological symptoms. The 2016 criteria further distinguish between two subtypes of seronegative AE: definite autoimmune LE and autoantibody-negative but probable AE (ANPRA).

In our case, a 43-year-old woman presented with refractory seizures and cognitive impairment. MRI findings of bilateral hippocampal and frontal T2/FLAIR hyperintensities suggested a limbic encephalitis-like picture, although CSF antibody testing was negative. Despite the lack of serological confirmation, the patient's response to IVMP and rituximab strongly supported an autoimmune etiology. The absence of specific biomarkers in SNAE poses diagnostic and therapeutic challenges often requiring empiric immunotherapy based on clinical and radiological features, as described by van Steenhoven and Titulaer [19]. As demonstrated in our patient, sustained seizure remission highlights the value of prompt intervention in managing such cases effectively.

Paraneoplastic autoimmune encephalitis

Paraneoplastic autoimmune encephalitis (PAE) is a subtype of AE associated with underlying malignancies, such as small cell lung cancer, ovarian teratoma, or testicular germ cell tumors. It is frequently linked to antibodies targeting intracellular antigens (e.g., anti-Hu and anti-Ma2) or neuronal surface proteins (e.g., anti-NMDA). The presence of an occult malignancy should always be suspected in cases of rapidly progressive encephalitis with unexplained neurological symptoms.

The diagnostic workup of PAE involves comprehensive imaging to identify an underlying malignancy. Our patient, a 28-year-old man, presented with fever, altered sensorium, recurrent seizures, and respiratory distress. MRI revealed left temporal and occipital T2/FLAIR hyperintensities, and subsequent imaging identified lung adenocarcinoma, confirming the paraneoplastic etiology. Management of PAE requires both immunotherapy (corticosteroids, IVIG, and rituximab) and aggressive treatment of the underlying malignancy, as described by Greenlee et al. [20]. Unfortunately, our patient succumbed to death due to oncological complications within four months, highlighting the poor prognosis often associated with PAE.

Final summary

Autoimmune encephalitis presents a diverse range of clinical manifestations that can mimic psychiatric disorders, viral encephalitis, and neurodegenerative diseases, often leading to diagnostic delays. In some cases, the lack of specific biomarkers further complicates early diagnosis and timely treatment initiation. In resource-limited settings, the unavailability of specific antibody panels poses a significant challenge, making it difficult to confirm autoimmune encephalitis and delaying immunotherapy administration. Additionally, while many patients respond well to immunotherapy, some develop treatment-refractory disease, requiring second-line immunosuppressants and prolonged rehabilitation.

Long-term follow-up is essential due to the risk of relapse, cognitive deficits, and psychiatric sequelae. Our case series highlights the heterogeneity of clinical outcomes, underscoring the necessity for multidisciplinary care and sustained rehabilitation. Despite advancements in the understanding and management of AE, the lack of standardized diagnostic criteria, particularly for seronegative cases, remains a significant limitation. The variability in clinical presentation, the absence of definitive biomarkers in some patients, and the reliance on clinical judgment pose challenges in ensuring early and accurate diagnosis.

## Conclusions

This case series underscores the diverse clinical presentations and diagnostic challenges associated with encephalitis, emphasizing the need for early recognition and intervention. The heterogeneity of symptoms, ranging from psychiatric disturbances to refractory seizures, necessitates a high index of suspicion and a multidisciplinary approach to management. Advanced neuroimaging, CSF analysis, and autoantibody testing are crucial in confirming the diagnosis, particularly in seronegative cases where clinical correlation remains essential.

Early initiation of immunosuppressive therapy, including corticosteroids, IVIG, plasma exchange, and rituximab, has been shown to improve outcomes and reduce the risk of long-term neurological deficits. However, treatment resistance and relapse remain concerns, underscoring the need for long-term follow-up and rehabilitation. Paraneoplastic cases further highlight the importance of malignancy screening in atypical presentations. Future research should focus on refining diagnostic criteria and exploring novel therapeutic strategies to enhance patient outcomes in autoimmune encephalitis.
